# Neurosyphilis: A Simulation Case for Emergency Medicine Residents

**DOI:** 10.7759/cureus.2984

**Published:** 2018-07-16

**Authors:** Chana Rich, Dimitrios Papanagnou, David Curley, Xiao Chi Zhang

**Affiliations:** 1 Emergency Medicine, Alpert Medical School of Brown University , Providence, USA; 2 Emergency Medicine, Thomas Jefferson University, Philadelphia, USA; 3 Emergency Medicine, Alpert Medical School of Brown University, Providence, USA

**Keywords:** neurosyphilis, emergency medicine, infectious diseases

## Abstract

Neurosyphilis is a dangerous and increasingly more prevalent sexually transmitted infection of the central nervous system caused by the bacterium *Treponema pallidum* that can present during the advanced stages of the disease (tertiary syphilis). Health care providers must remain vigilant in screening for syphilis in patients with high-risk behaviors as a delay in diagnosis and treatment may lead to symptom progression and debilitating sequelae years later. To date, there have been no published simulation case studies on neurosyphilis. This simulation case, based on a real patient encounter, is written for emergency medicine residents to diagnose and manage a patient presenting with the sequelae of neurosyphilis. This case was run for four separate iterations at a simulation center with two residents and an attending physician acting as confederates. Following the case, learners were provided with bedside debriefing, and a question and answer session. Based on post-simulation qualitative assessment, junior residents alone were less likely to perform a comprehensive integumentary exam without the presence of senior residents, although both groups failed to elicit pertinent sexual history until they discovered syphilitic lesions. After case completion and debriefing, all learners were able to demonstrate the understanding of the primary learning objectives.

## Introduction

Syphilis is a sexually-transmitted infection (STI), spread by the bacterium Treponema pallidum (T. pallidum). The global incidence rate is 1.5 cases per 1000 females and 1.5 cases per 1000 males [1-2]. Syphilis is divided into four stages (i.e., primary, secondary, latent, and tertiary), each with associated signs and symptoms. The disease course is characterized by episodes of active clinical disease with periods of latent infection. Although the clinical course is divided into stages, there is overlap between the stages and patients may not recollect signs of an earlier disease stage [3].

T. pallidum infection occurs when the bacterium gains access to subcutaneous tissue through microscopic abrasions [2]. During the primary stage of syphilis, a patient may notice a single lesion or multiple lesions at the site of entry. Sores are round and painless, and usually last for several weeks [4]. During the secondary stage, a patient may develop a rash over one or more areas of the body. The patient also develops red or brown spots on the palms of the hands and/or soles of the feet. Only one-third of patients go on to develop tertiary syphilis [5]. Tertiary syphilis can affect many different organ systems, including the cardiovascular system and the brain. During the latent stage of syphilis, there are no symptoms; however, serum tests may still be positive for infection.

Not all patients with T. pallidum in the cerebrospinal fluid (CSF) develop symptomatic neurosyphilis, as many cases resolve by the end of the secondary stage of the disease [3]. However, patients who are untreated may develop symptomatic neurosyphilis in four to nine percent of the time [3]. Prior to the advent of antibiotics, neurosyphilis was seen in a quarter to a third of syphilis patients; however, this is now significantly less frequent [6]. Today, neurosyphilis is more frequently seen in patients with human immunodeficiency virus infection (HIV) [7]. Neurosyphilis can occur during any stage of the disease [3]. Approximately one-quarter of patients with syphilis have *T. pallidum* in the CSF [8-9].

Neurosyphilis can be divided into early and late forms. Early neurosyphilis affects the CSF, meninges, and vasculature, while late forms affect the brain and spinal cord parenchyma [10]. Patients with early symptomatic neurosyphilis may complain of cranial nerve neuropathies, headache, nausea, vomiting, stiff neck, or confusion. Additional neurovascular complications include infectious arteritis in the brain or spinal cord, which can present with stroke-like features. Ocular syphilis may also be present in early neurosyphilis and may involve almost any eye structure. Posterior uveitis and panuveitis are the most common, and patients present with decreased visual acuity [11]. Late findings of neurosyphilis include general paresis and tabes dorsalis. General paresis is a progressive dementing illness that can develop up to 25 years after primary infection [10]. Early on, patients may experience forgetfulness or personality changes; however as time progresses, memory loss, altered judgment, and severe dementia ensue. Patients with tabes dorsalis, or locomotor ataxia, may have pupillary abnormalities, including Argyll-Robertson pupils, progressive ataxia, and bowel and bladder dysfunction [10].

Tabes dorsalis is a disease of the posterior columns of the spinal cord and dorsal roots, in which patients may experience sensory ataxia, sudden, brief, stabbing pain, absent lower extremity reflexes, impaired vibratory or positional sensation. Tabes dorsalis is often associated with Argyll-Robertson pupil, an ocular pathognomonic feature in which the affected pupil accommodates, but does not react to light stimulation.

The diagnosis of neurosyphilis is based on clinical exam and examination of the blood and cerebrospinal fluid. Serum can be tested for syphilis using treponemal tests, such as fluorescent treponemal antibody absorption (FTA-ABS) or nontreponemal tests, such as Venereal Disease Research Laboratory test (VDRL) and Rapid Plasma Reagin (RPR). Traditional algorithms for diagnosing syphilis involve initial screening with RPR followed by confirmation with a treponemal test, such as FTA-ABS [12]. Treponemal tests are often reactive in neurosyphilis; however, nontreponemal tests are not as reliable in late neurosyphilis. Serum VDRL may be negative in up to 25 percent of patients with neurosyphilis. The CSF of a patient with tabes dorsalis may be normal or show only mild pleocytosis and protein elevation.

CSF abnormalities vary depending on the stage of the disease. Lumbar puncture is recommended in patients who present with neurologic or ocular disease that could be caused by syphilis, and in patients with HIV co-infection with syphilis at any stage [12]. CSF VDRL is very specific; however, the sensitivity ranges from only 30%-70% [3]. CSF VDRL is preferred over CSF RPR for the diagnosis of neurosyphilis [12]. CSF treponemal antibody tests are sensitive, but not specific; therefore, if the clinical suspicion for neurosyphilis is high, it cannot be used to rule out the diagnosis [13] Common CSF findings in patients with neurosyphilis include mild mononuclear pleocytosis and elevated protein.

The treatment of early syphilis is typically a single dose of penicillin G benzathine, 2.4 million units intramuscularly (IM). Patients with late syphilis, tertiary syphilis or late latent syphilis may be treated with 2.4 million units of IM penicillin G benzathine once weekly for three weeks. The Center for Disease Control and Prevention recommends one of two regimens for the treatment of neurosyphilis [12]. The first regimen is three to four million units of penicillin G intravenously every four hours for 10 to 14 days. The alternative regimen is 2.4 million units of intramuscular procaine penicillin G daily plus 500 mg of probenecid orally four times a day for 10 to 14 days. If the patient has a mild penicillin allergy, re-challenging the patient or desensitization is recommended. Otherwise, intravenous or intramuscular ceftriaxone can be used. Ocular syphilis or otosyphilis are treated with the same regimen, regardless of CSF results. The Jarisch-Herxheimer reaction (i.e., acute febrile reaction) may occur within the first 24 hours of penicillin treatment for syphilis.

## Technical report

Methods

This simulation case is based on a fictional scenario of neurosyphilis in a patient who presents to the urgent care with persistent neurological symptoms and concealed dermatologic findings. The simulated case was based on a real patient encounter and designed by a panel consisting of an emergency medicine (EM) attending and two EM residents to allow learners to clinically diagnose an unusual, but high-risk, disease process (i.e.,neurosyphilis) through a combination of insightful history taking and detailed physical exam findings, while learning how to communicate amongst team members, consultants, and the patient in a safe, simulated environment. A Sim Man 3G was incorporated into the simulation case to carefully conceal certain revealing aspects of the history and/or physical exam (per the script) to provide appropriate pathologic pupillary reflexes for heightened realism to the case.

Equipment

**Figure 1:**
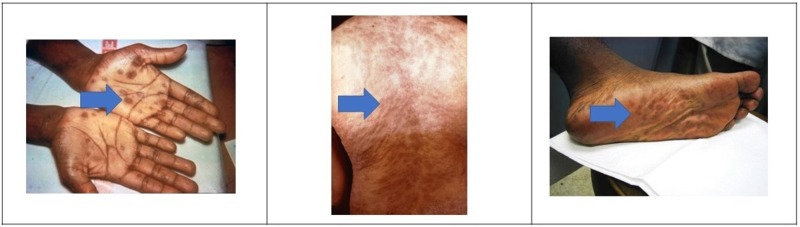
Secondary Syphilis Lesions Syphilis rash can appear as red or reddish brown spots on the hands, feet, or trunk (arrows).

1.       Sim Lab Set Up: A room simulating a typical urgent care room (with a stretcher, IV pole) with an adjacent room equipped with audio-visual capabilities, and monitor, with another adjacent room for case debriefing)

2.       Sim Man 3G Set Up: winter gear with gloves

3.       Moulage: Small pink dots (washable marker) on trunk, feet, and hands

4.       Images: Syphilis rash (Figure 1) [14].

Personnel/Roles:

1.        Nurse - Confederate role (i.e., played by a resident, fellow, or attending)

2.       Patient - Mannequin (Voice by either a resident, fellow, or attending) or patient actor

3.       Receiving physician - Resident, fellow, or attending (over the phone)

**Figure 2:**
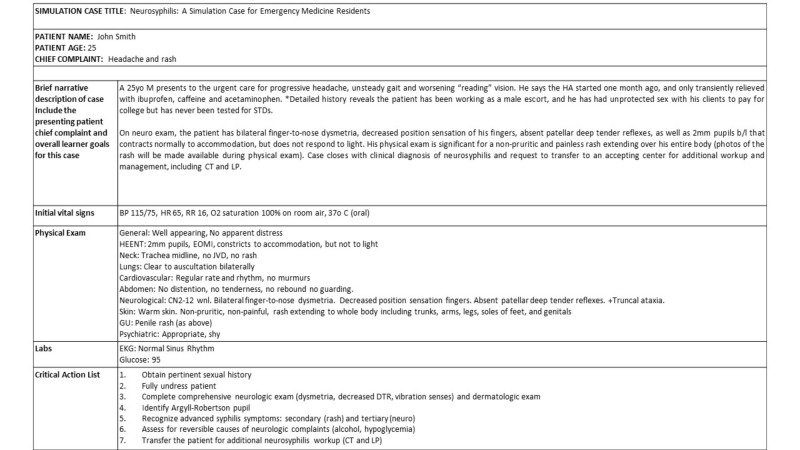
Neurosyphilis Case Flow

Implementation:

Before the case begins, facilitators are advised to first establish the roles of each confederate and provide a preview of the flow of case (Figure 2). All available laboratory tests should be provided to the learners upon request. The case begins with the learners meeting the patient at the bedside. Following the simulation, the participants should complete a bed-side debriefing.

Assessment:

Learners will be assessed based on their active participation in both the case scenario and case debriefing. The debriefing will be separated into two components: 1) bedside teaching; and 2) question-and-answer format. 

Bedside Teaching:

At the end of the case, the instructor(s)/debriefer(s) should gather information about the delegation of roles and responsibilities while eliciting a case summary from the team leader. The instructor(s) can also request supplemental case information and participant performance, specifically strengths and opportunities for improvement from the team members or observers (if available). The instructor(s) should review the critical action checklist, which includes questions and emphasizes the importance of gathering pertinent patient information and exposing patients for a complete physical examination.

Question-and-Answer Session:

All learners are encouraged to participate. The debriefing session concludes with each participant sharing one take-home message he/she drew from the case discussion with the rest of the group.

Results:

Based on qualitative assessment, investigators found that both junior and senior residents were unlikely to elicit a pertinent sexual history or perform a comprehensive integumentary exam on the patient during the simulated case. Junior residents alone were even less likely to fully remove the patient's clothing to assess for additional lesions without the presence of senior residents, although both groups failed to elicit a pertinent sexual history until they discovered syphilitic lesions. Groups consisting of junior residents required additional prompts from both the patient and the nurse to volunteer additional information (i.e. the nurse would mention the patient’s rash or ask about a significant other). Both groups planned on transferring the patient to an emergency department capable of performing a lumbar puncture for further testing.

It was also noted that while all learners evaluated for pupillary findings after the patient described vision changes, no one recognized the affective pupillary defect until either the patient or the confederate asked the learners to carefully examine each pupil’s reaction to the light from each light stimulus.

Prior to patient transfer, both groups were able to correctly explain the basic pathophysiology for syphilis and neurosyphilis to the patient in lay-person terms. Throughout the debriefing (i.e., open-ended questions and “take-home-message” summarization), learners demonstrated an understanding of the learning objectives of the case: to identify the symptoms of neurosyphilis; to assess for STI risk factors; to recognizing the need for a patient transfer to an ED if there is a concern for neurosyphilis; and to educate patients on safe-sex practices.

## Discussion

“Neurosyphilis: A Simulation Case for Emergency Medicine Residents” is based on a fictional scenario of neurosyphilis in a patient who presents to the urgent care with persistent neurological symptoms and concealed dermatologic findings. Medical simulation has become an invaluable platform for medical education by allowing learners to be exposed to rare and fatal disease processes in a targeted, simulated learning environment. After a thorough review through the MedEdPORTAL database, the investigators did not find any cases or educational modules discussing neurosyphilis. This simulation case afforded learners the opportunity to clinically diagnose neurosyphilis.

Specifically, learners practiced the skills needed to correctly identify, manage, and treat neurosyphilis. Additionally, learners had to recognize the need for further testing and transfer to an ED capable of further testing. After four separate case iterations, the investigators noted that both junior and senior EM residents were unlikely to elicit a pertinent sexual history or fully expose the patient during the simulated case without prompting. The investigators also noted that the junior residents had a greater knowledge gap than senior residents in terms of acute management of neurosyphillis and transfer indications. Overall, the investigators noted the case worked well for both junior and senior EM residents. After prompting, residents endorsed that they appreciated the concealed elements of the case (i.e., rash and sexual history).

This simulated case was deliberatey designed to take place in a practice setting that necessitated a transfer for further patient work-up. This further added to the educational objectives of the case: to recognize when a patient requires diagnostic testing or procedural intervention(s) that are not available in a specific clinical setting.

One of the most challenging and crucial components for this simulation case was identifying the unique pupillary changes associated with neurosyphilis. Residents are typically unfamiliar with high-fidelity models and their capability to reproduce detailed, dynamic pupillary exam findings. Despite real-time manual pupillary adjustments [performed by the instructor] in concert with the residents’ exam, many residents simply shined a light in the simulator's eye and asked what they saw or if they were equal; they had to be prompted that the exam was the actual exam. This prompted the investigators to consider including a specific pre-brief describing the unique capabilities of the high-fidelity models (i.e. blink, pupillary dilation, sweat) to encourage learners to suspend their disbelief and trust their exam findings, as opposed to constantly asking the instructor for confirmation of exam findings.

We recognize the limitations to “Neurosyphilis: A Simulation Case for Emergency Medicine Residents:” there were a small number of participants from a single program. As the case was also presented as a training session, there were no control groups. Moreover, results were observational. Given how uncommon this diagnosis is, however, our case provides EM learners the opportunity to diagnose and manage neurosyphilis.

## Conclusions

Future educational opportunities include running a similar case in the emergency department setting, rather than in an outpatient clinical setting. This change in practice setting would enable residents to practice the lumbar puncture procedure. There are multiple other early and late sequelae of syphilis, such as general paresis and tabes dorsalis, that could also be incorporated into future cases.
